# (CO_2_)_n_^+^, (H_2_O)_n_^+^, and (H_2_O)_n_^+^ (CO_2_) gas cluster ion beam secondary ion mass spectrometry: analysis of lipid extracts, cells, and Alzheimer’s model mouse brain tissue

**DOI:** 10.1007/s00216-021-03372-x

**Published:** 2021-05-11

**Authors:** Kelly Dimovska Nilsson, Anthi Karagianni, Ibrahim Kaya, Marcus Henricsson, John S. Fletcher

**Affiliations:** 1 Present Address: Department of Chemistry and Molecular Biology, University of Gothenburg, 405 30 Gothenburg, Sweden; 2 Department of Psychiatry and Neurochemistry, Sahlgrenska Academy at the University of Gothenburg, 413 45 Mölndal, Sweden; 3 Medical Mass Spectrometry Laboratory, Department of Pharmaceutical Biosciences, Uppsala University, 751 05 Uppsala, Sweden; 4 Department of Molecular and Clinical Medicine/Wallenberg Laboratory, Institute of Medicine, University of Gothenburg, 41345 Gothenburg, Sweden

**Keywords:** SIMS, Alzheimer’s, Lipids, Imaging, Mass spectrometry, Water clusters

## Abstract

This work assesses the potential of new water cluster-based ion beams for improving the capabilities of secondary ion mass spectrometry (SIMS) for in situ lipidomics. The effect of water clusters was compared to carbon dioxide clusters, along with the effect of using pure water clusters compared to mixed water and carbon dioxide clusters. A signal increase was found when using pure water clusters. However, when analyzing cells, a more substantial signal increase was found in positive ion mode when the water clusters also contained carbon dioxide, suggesting that additional reactions are in play. The effects of using a water primary ion beam on a more complex sample were investigated by analyzing brain tissue from an Alzheimer’s disease transgenic mouse model. The results indicate that the ToF-SIMS results are approaching those from MALDI as ToF-SIMS was able to image lyso-phosphocholine (LPC) lipids, a lipid class that for a long time has eluded detection during SIMS analyses. Gangliosides, sulfatides, and cholesterol were also imaged.

**Supplementary Information:**

The online version contains supplementary material available at 10.1007/s00216-021-03372-x.

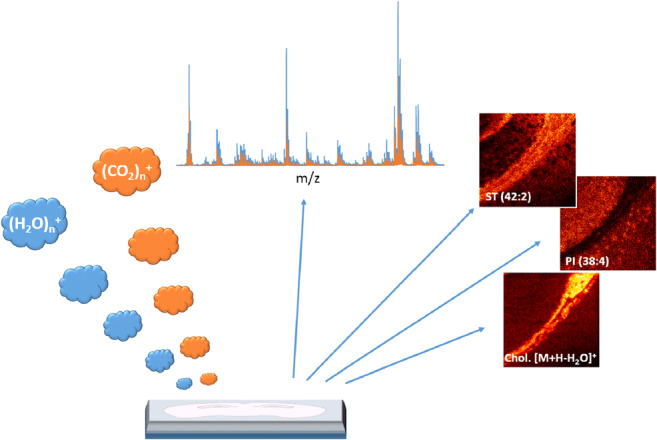

## Introduction

Secondary ion mass spectrometry (SIMS) is one of the increasing number of imaging mass spectrometry approaches that combine the chemical specificity of mass spectrometry with imaging capabilities that in extreme cases can approach 10-nm lateral resolution [1–3].

Different imaging mass spectrometry approaches (e.g., MALDI or DESI) offer different advantages such as intact protein detection or ambient analysis [4, 5]. SIMS is unique in the ability to finely focus, and electrostatically scan, the ion beam used to probe the sample and also deliver information with high surface sensitivity and high depth resolution, if the ion beam is used to gradually erode the sample during the analysis.

While SIMS has been used in a wide range of research fields for the analysis of both organic and inorganic samples, the area of biological analysis is one of the most rapidly expanding. Progress has been greatly aided by the development of a range of different ion beams that can be fired at the sample to generate the secondary ions that are extracted and analyzed in the mass spectrometer. Metal cluster ion beams such as Au_3_^+^ and Bi_3_^+^ have supplanted the traditional monoatomic ion beams (e.g., Ga^+^), as they provide non-linear enhancements of secondary ion signal, as the cluster size increases while still being relatively easy to focus [6, 7]. Very large, high energy gold clusters (e.g., Au_2800_^8+^) have also been shown to enhance *pseudo-*molecular ion detection due to lower energy sputtering of secondary species [8].

Polyatomic ion beams such as C_60_^+^ and more recently gas cluster ion beams (GCIBs) that typically contain several thousand atoms (e.g., Ar_2000_^+^) offer particular benefits for the detection of intact molecular and *pseudo*-molecular ions [9, 10]. The GCIBs have become widespread for etching organic samples for depth profile analysis both in SIMS and X-ray photoelectron spectroscopy (XPS). Additionally, early work on amino acids and polymer samples showed that at low energy per atom, the cluster beams reduced fragmentation evidenced in the SIMS spectra [11]. Such a reduction in fragmentation is considered to be a benefit for biological SIMS despite inspection of fragmentation patterns and fragment ion co-localization being tools for lipid or protein identification [12–14].

A drawback of the GCIBs is that it is very difficult to produce very short pulses of ions from the relatively slow-moving projectiles that normally have a range of sizes, perhaps ± 1000 atoms. As the mass resolution of conventional ToF-SIMS instruments is dependent of the pulsing of the primary ion beam, the majority of examples of GCIBs as analysis beams for SIMS have used more recent spectrometer configurations, such as the buncher-ToF combination of the J105 instrument from Ionoptika and more recently the Orbitrap-SIMS instrument from IONTOF [15, 16].

Angerer et al. compared the secondary ion signals for different lipids in rat brain tissue using equivalent energy (40 keV) C_60_^+^ and Ar_4000_^+^ ion beams and reported enhancements between × 30 and × 50 for intact phospholipid and ganglioside peaks [17]. Improved focusing facilitated by higher energy (70 keV) beams has been used for 3D single-cell imaging with GCIBs [18].

While these advances are significant and have been exploited to good effect in a range of studies, often focused on in situ lipidomics, the benefits come from reduced fragmentation and not an increase in ionization [16, 19, 20]. Decreasing the energy/atom in the gas cluster below 5 eV may result in gentler sputtering of material and less fragmentation, but at a detriment to secondary ion formation and hence mass spectrometric sensitivity. Significantly, increasing the ionization efficiency, which for many biological samples is estimated to be well below 1%, possibly around 1 × 10^−5^, remains the holy grail for biological SIMS analysis. Many methods have been employed, with varying degrees of success, to enhance ionization in SIMS ranging from matrix addition, metal addition, and chemical exposure including derivatization [21–26]. Water as ice in frozen samples or vapor applied close to the sample surface has also been shown to be beneficial [27–29]. Analysis of frozen hydrated biological samples has also been shown to improve secondary ion yields [30–32].

One approach suggested for reaching this goal is through the use of reactive cluster ion beams. For biological analysis, several proof of principle papers have been published highlighting the potential of using water cluster beams of the type (H_2_O)_n_^+^ [33–37]. The use of the water cluster was initially envisaged as a proton source that might aid the formation of [M + H]^+^ ions due to the interaction with the primary particle with the sample and the target analyte during the ejection process. Indeed, significant enhancement of different secondary ion species has been reported with the highest enhancements being for [M + H]^+^ ions but enhancements have also been reported for cationized species such as [M + Na]^+^ and [M + K]^+^ ions, and also [M-H]^−^ ions in negative ion mode analysis.

In this paper, we report the effects of different water clusters on lipid signals and that the use of CO_2_ as a backing gas for the water cluster ion beam in some cases provides additional signal enhancement. Additionally, the use of the new beams allows lysophospholipids to be imaged in amyloid plaques in coronal brain tissue sections of familial Alzheimer’s transgenic mouse model, (5 × FAD) for the first time using ToF-SIMS.

## Materials and methods

### Sample preparation

#### Lipid extracts

Droplets of lipid extracts in chloroform from porcine brain and bovine heart (Avanti Polar Lipids Inc. purchased from Merck) were spotted onto silicon wafers and left to air dry before ToF-SIMS analysis.

Within the droplet area, no substrate patches were visible (and no substrate signals were detected in the MS analysis) providing confidence that the number of molecules analyzed in each region within the different droplets was similar.

#### Cells

MCF7 cells, a breast cancer cell line, were purchased from American Type Culture Collection (ATCC) and incubated in Dulbecco’s modified Eagle’s medium (DMEM) supplemented with 10% fetal bovine serum, 1% penicillin/streptomycin, 1% L-glutamine (all from Thermo Fisher Scientific), and 1% MEM non-essential amino acids (Sigma-Aldrich). The cells were incubated in individual wells containing pre-cleaned Si wafers (1 cm × 1 cm) at 37 °C with 5% CO_2_ in humidified atmosphere. The cells were incubated for approximately 24 h before 16.7 μL of ethanol (99.7%) was added to the cell media. The cells were then left to incubate for another 24 h before the wafers were washed in ammonium formate solution (0.15 M) three times, frozen in liquid nitrogen, and then stored in − 80 °C until ToF-SIMS analysis. Before the ToF-SIMS analysis, the cells were freeze-dried inside the ToF-SIMS instrument.

#### Mouse brain tissue

Mouse brain tissue from a 5xFAD transgenic mouse model was purchased from Jackson Laboratory (Bar Harbor, ME). Mice were anesthetized using isoflurane and sacrificed by decapitation. The mouse brains were removed with less than a 3-min postmortem delay and then frozen on dry ice. The tissue was cut into 10-μm-thick sections at − 20 °C using a Leica cryostat microtome. The tissue sections were collected onto indium tin oxide (ITO)-coated glass slides and stored at − 80 °C until analysis. Amyloid plaque staining was performed as described previously [38] using rabbit polyclonal antibody against β-amyloid (Abcam ab2539, Cambridge, UK) with anti-rabbit Ig (Vector Laboratories, CA, USA), as secondary reagent and visualized using Liquid DAB+ substrate chromogen system (DAKO). The cell nuclei were counter stained using hematoxylin. Prior to the ToF-SIMS analysis, the tissue sections were freeze-dried for 30 min.

### ESI-MS analysis

To acquire high-resolution full spectra, extracts from porcine brain and bovine heart were infused into a Synapt G2 instrument (Waters, Manchester, UK) in both positive and negative ion mode. The extracts were diluted × 20 (for negative mode) or × 200 (for positive mode) and infused using a syringe pump at 50 μL/min. Full scan spectra were acquired during 1 min (at 1 scan/s) in continuum mode.

For relative quantitation of phosphatidylethanolamines and phosphatidylcholines in positive ion mode, the extracts were diluted in chloroform:methanol [1:1] with 5 mM ammonium acetate and spiked with diheptadecanoin (C17:0)-containing phosphatidylcholine and phosphatidylethanolamine. The extracts were infused into a QTRAP 5500 mass spectrometer (Sciex, Concord, Canada) equipped with a robotic nanoflow ion source, TriVersa NanoMate (Advion BioSciences, Ithaca, NJ). Phosphatidylcholines were detected using precursor ion scanning for *m/z* 184 and phosphatidylethanolamines were detected using neutral loss for *m/z* 141.

### ToF-SIMS analysis

ToF-SIMS analyses were performed using a J105-*3D Chemical Imager* (Ionoptika Ltd., UK). This instrument has been described in detail elsewhere [15]. In brief, this is a ToF-SIMS instrument which uses a quasi-continuous primary ion beam which allows for use of large clusters as primary ion projectile. The J105 instrument utilizes a linear buncher which compresses the stream of secondary ions and thereby creates a time focus as the ions enter the time-of-flight analyzer. This makes the mass spectra generated by this instrument less sensitive to topographical differences and charging.

The instrument used was equipped with a 40 kV GCIB (Ionoptika Ltd.) fitted with a water cluster ion source first described by Sheraz et al. [34, 39]. In short, water vapor is generated in a temperature-controlled water reservoir fitted before the expansion chamber. To prevent water condensation, the water vapor subsequently passes through a heated nozzle into the expansion chamber where neutral clusters are formed through adiabatic expansion. The neutral clusters are later ionized by electron impact. In this study, three types of clusters were used as primary ion beams, CO_2_ and H_2_O and H_2_O clusters generated with a back pressure of CO_2_ that are expected to incorporate some CO_2_ into the cluster. The clusters are labeled (CO_2_)_n_^+^, (H_2_O)_n_^+^, and (H_2_O)_n_^+^(CO_2_), respectively.

The effect of cluster size (e.g., energy/nucleon) was investigated by analyzing lipid extract droplets where the cluster size was varied between 6000 and 25,000 molecules to find the optimal cluster size for the rest of the study. Analysis of lipid extracts was performed over the mass range *m/z* 60–3000 with a fluence of 2.56 × 10^12^ ions/cm^2^.

To study the effect on lipid distribution and signal intensity using (H_2_O)_n_^+^ clusters as the primary ion projectile compared to using (CO_2_)_n_^+^, three lipid droplets were analyzed with (CO_2_)_6k_^+^ and (H_2_O)_22.5k_^+^ over the mass range *m/z* 60–3000 with a fluence of 2.54 × 10^12^ ions/cm^3^.

To investigate the effect of using pure (H_2_O)_n_^+^ clusters compared to clusters of (H_2_O)_n_^+^ doped with CO_2_ on secondary ion yield and matrix effects, analyses of lipid extracts were performed with two different backing gasses, CO_2_ and N_2_. N_2_ was chosen since N_2_ does not readily form clusters; hence, we can assume that the clusters would consist purely of water. As a comparison, the same lipid extracts were also analyzed with (CO_2_)_6k_^+^.

Cell analysis of a MCF7 cell line was performed to test the effect of using different water clusters on a more complex sample. Cell analysis was performed using (CO_2_)_7k_^+^, (H_2_O)_18k_^+^, and (H_2_O)_18k_^+^ (CO_2_) as primary ion projectiles in the mass range *m/z* 60–1000 with a fluence of 1.02 × 10^13^ ions/cm^2^.

Finally, tissue analysis was performed on mouse brain using (CO_2_)_7k_^+^ and (H_2_O)_18k_^+^ (CO_2_) as primary ion projectiles in the mass range *m/z* 110–3000 with a fluence of 2.1 × 10^12^ ions/cm^2^ and 7.6 × 10^12^ ions/cm^2^, respectively.

Peak assignments mentioned in this work are putatively assigned based on mass accuracy and isotopic distribution and are presented in Table S1 in the Supplementary Information (ESM).

### Data analysis

**Fig. 1:**
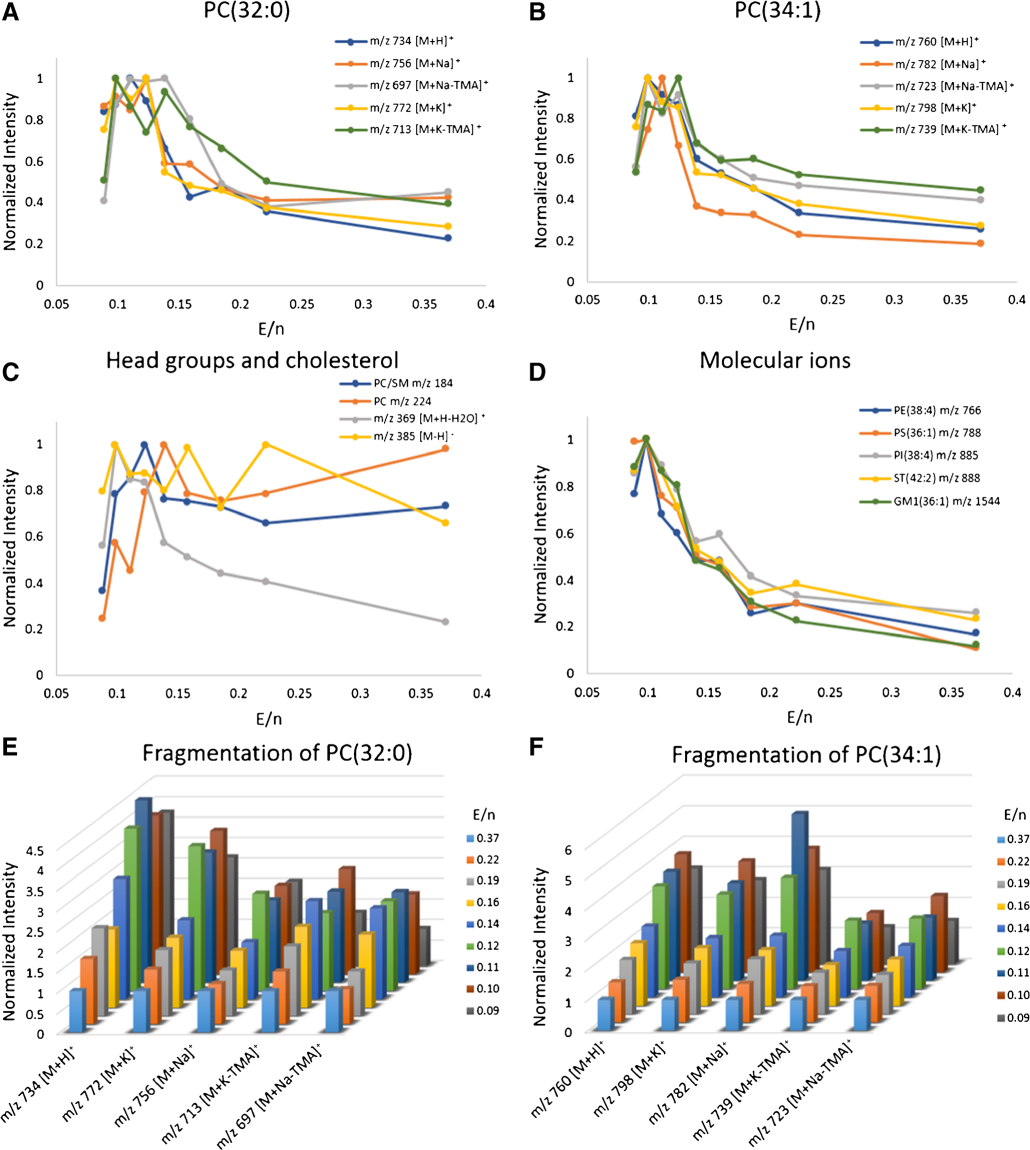
**A**–**D** Plots of various ion species from analysis of porcine brain lipid extract showing signal intensity versus E/n as the cluster size varies from 6000 to 25,000. Signal normalized to the highest signal. Characteristic high mass ions from two phosphatidylcholine (PC) lipids, PC(32:0) and PC(34:1), are shown in **A** and **B**, respectively. Head group ions from PC lipids are shown in **C** along with ions associated with cholesterol. [M-H]^−^ ions detected in negative ion mode are presented in **D**. 3D bar charts showing reduced fragmentation of various species with lower E/n are presented in **E **and **F**. Signal normalized to the signal when using cluster size 6000. Analysis area 200 μm × 200 μm with a fluence of 2.56 × 10^12^ ions/cm^2^

**Fig. 2:**
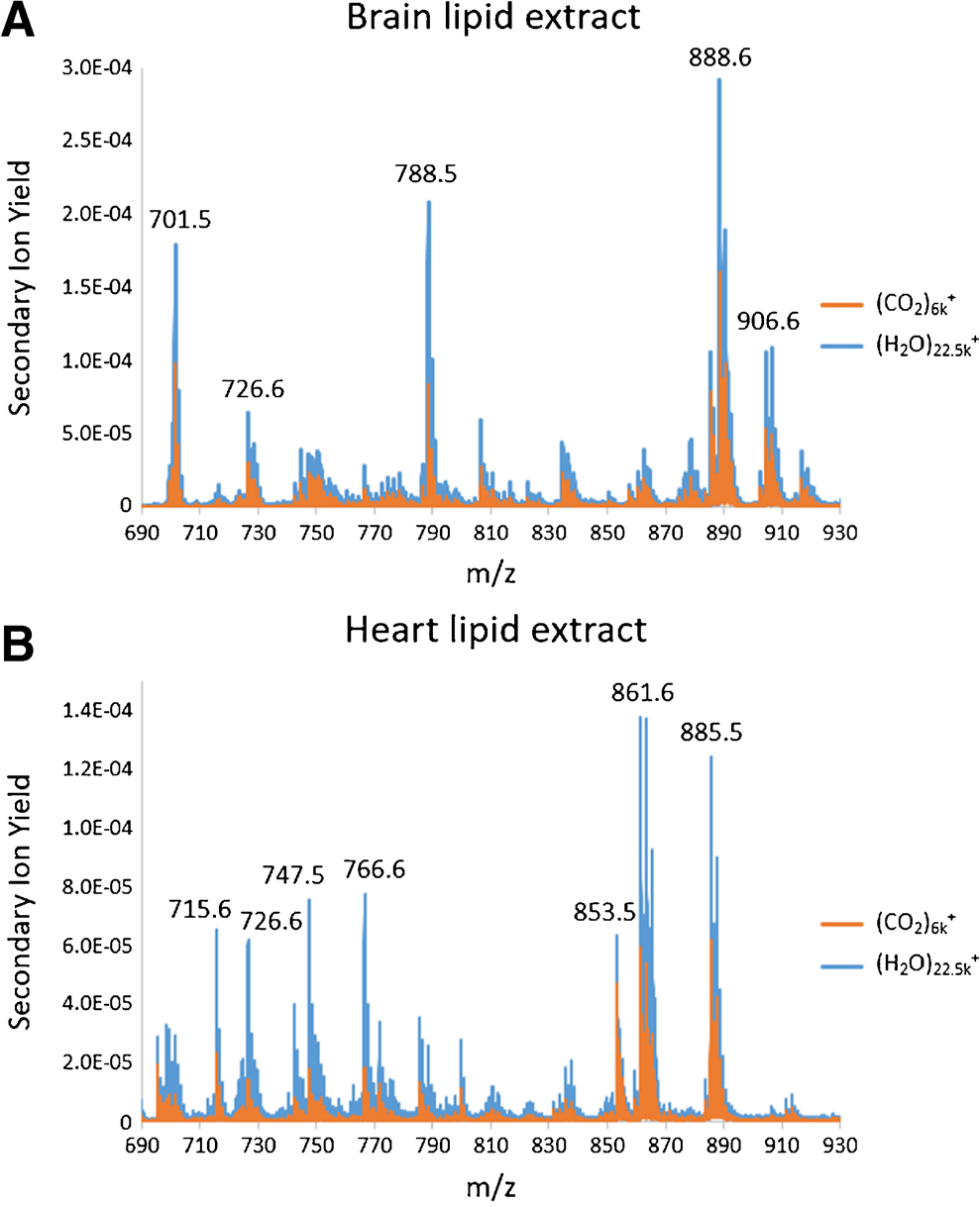
Excerpts of overlayed mass spectra of lipid extracts acquired in negative ion mode. **A** Porcine brain lipid extract. **B** Bovine heart lipid extract. The samples were analyzed using two different primary ion beams, (H_2_O)_22.5k_^+^ and (CO_2_)_6k_^+^. Analysis area 233 μm × 233 μm with a fluence of 2.54 × 10^12^ ions/cm^2^

**Fig. 3:**
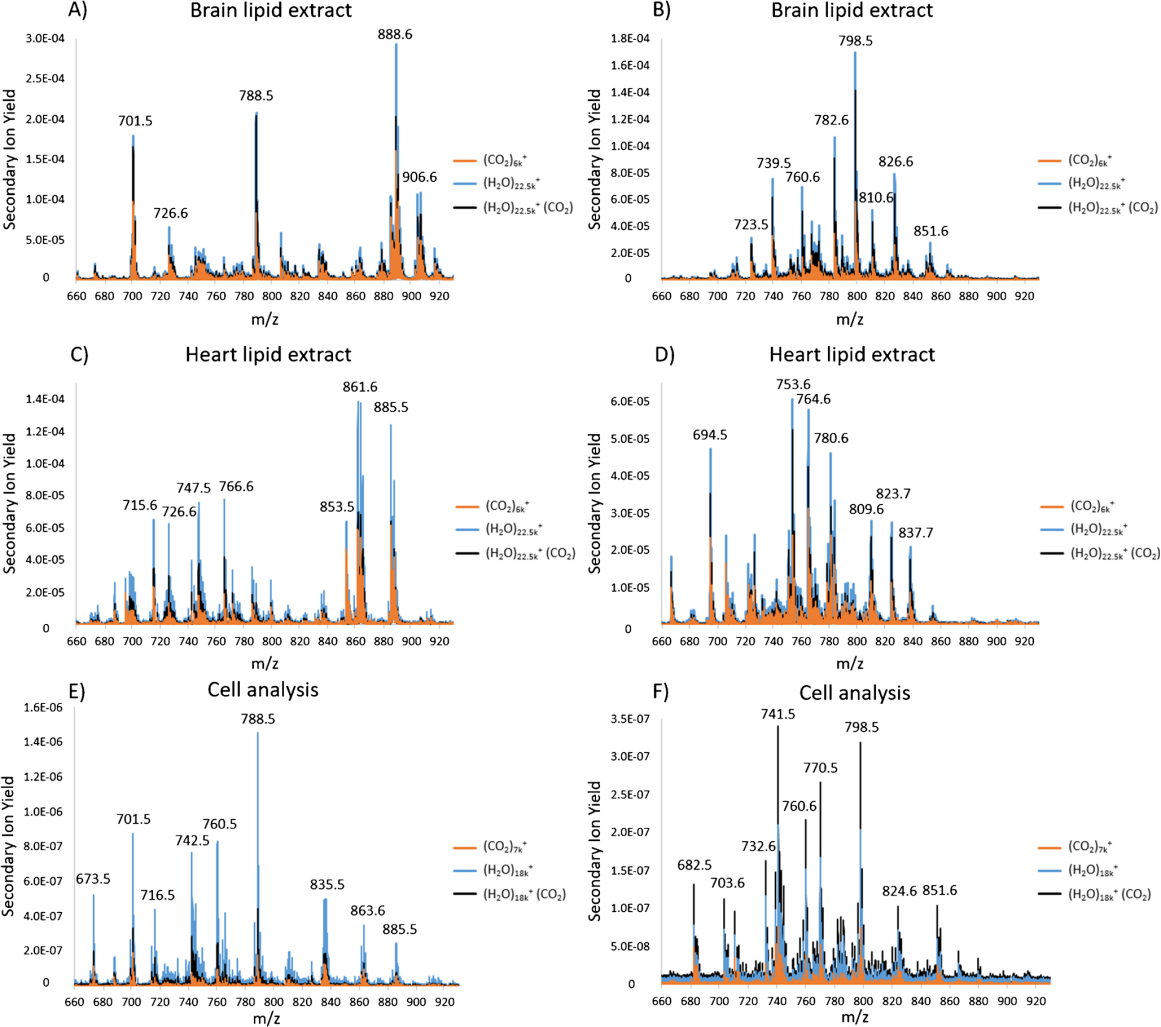
Spectral overlays of excerpts from spectra of **A**, **B** porcine brain lipid extract, **C**, **D** bovine heart lipid extract, and **E**, **F** cells acquired with three different primary ion projectiles; (CO_2_)_7k_^+^, (H_2_O)_18k_^+^, and (H_2_O)_18k_^+^ (CO_2_). **A**, **C**, and **E** negative ion mode data. **B**, **D**, and **F** positive ion mode data. Lipid analysis area 233 μm × 233 μm with a fluence of 2.54 × 10^12^ ions/cm^2^. Cell analysis area 600 μm × 600 μm with a fluence of 1.02 × 10^13^ ions/cm^3^

Secondary ion spectra from lipid samples were extracted from ion images acquired within the droplet area. The data in Fig. 1 were normalized to the highest signal level to more easily visualize the trend in signal variation with cluster size. In Figs. 2 and 3, spectral overlays are displayed where the signal is plotted relative to the number of primary ions impacting the sample (counts/primary ion) generally referred to as secondary ion yield. It should be noted that the J105 instrument used in this study uses an analogue counting system, so multiple counts per secondary ion are recorded. Hence, care should be taken when comparing this data with that from instruments with different detection/counting systems.

Replicate lipid analyses were performed on three different lipid droplets. Error estimations on the enhancements include the variance of the replicate data and the precision of the primary ion current measurement (±0.5 pA).

Image data are displayed on a thermal color scale normalized to the maximum intensity in each individual image.

## Results and discussion

### Fragmentation pattern in lipid extracts

Initial experiments were performed on commercially available lipid extract samples using water vapor with a N_2_ backing gas to ensure pure water cluster formation. Lipids from porcine brain and bovine heart were analyzed in order to cover a broader range of lipid classes. Dried lipid droplets on silicon wafer substrates were analyzed using different sized water clusters ranging from 6000 to 25,000. All analyses were performed using a 40-kV primary ion accelerating potential so that the energy per nucleon (E/n) was dependent only on the cluster size. E/n, the energy divided by the number of protons and neutrons in the cluster, is often used as it allows simpler comparison between clusters that comprise different constituent atoms or molecules (e.g., CO_2_ or H_2_O in this study). In each case, the analysis was performed to the same fluence and using the same number of primary ions.

Figure 1 shows data from analysis of porcine brain lipid extract. The data displayed in Fig. 1A**–**D is plotted as a function of signal intensity versus E/n and is normalized to the maximum signal for each ion species to clearly depict the optimum cluster size for lipid analysis. Figure 1E–F show 3D bar charts where the data is also plotted as a function of signal intensity versus E/n, but the data is normalized to the signal intensity obtained with the smallest cluster (E/n = 0.37), again to clearly illustrate the optimum cluster size for lipid analysis. The plots in Fig. 1A, B, and D show that for the phospholipids analyzed, a signal increase is observed with larger cluster size, i.e., lower E/n. Equivalent figures with data from analysis of bovine heart lipid extract are found in the ESM (Fig. S1).

The signal maxima for [M + H]^+^ ions and large fragments from phosphatidylcholine (PC) lipids, PC(32:0) and PC(34:1), and the PC/sphingomyelin (SM) head group at *m/z* 184 are all in the interval between approximately E/n = 0.1 at cluster size 22.5 k and E/n = 0.14 at cluster size 16 k (Fig. 1A–C). The PC head group-associated ion at *m/z* 224 has two signal maxima, one at E/n = 0.37 at cluster size 6 k and one at E/n = 0.14 (Fig. 1C). A possible explanation for this is that the first signal maxima at E/n = 0.37 is due to more fragmentation which is reduced with larger clusters; however, the increase in cluster size is also expected to lead to an increase in signal intensity, which would explain the second signal maxima at E/n = 0.14. A similar trend is seen with *m/z* 184, although not as obvious. In the case of [Chol. + H-H_2_O]^+^ at *m/z* 369, the signal maximum is E/n = 0.1, while [Chol. − H]^−^ at *m/z* 385 on the other hand does not show a clear signal maximum (Fig. 1C). It should be noted that the signal for the *m/z* 385 peak for cholesterol is already very low even with the smallest of the water clusters used here. The reduction of the intensity of this ion compared to the [Chol. + H-H_2_O]^+^ ion at *m/z* 369 has previously been shown with Ar_n_^+^ clusters compared to C_60_^+^ ion beam analysis [17], suggesting that this ion is produced as a result of a more energetic process.

The signal maxima for the intact lipids acquired in negative ion mode are found at E/n = 0.1 eV (Fig. 1D). This is in good agreement with previous observations for [M-H]^−^ species of PC(34:1) and [M-H]^−^ species of a cardiolipin sodium salt at 40 keV impact energy, where the ion yield maxima were also reached around E/n = 0.1 [37]. These new results show that this appears to be a general trend for a wide range of lipid types. The signal maxima for ions in positive ion mode were less consistent over the molecular species and fragments, however, still in the range between E/n = 0.1 and 0.14 which again is in close agreement with previous observations mentioned above [37].

The increased secondary ion yield of *pseudo-*molecular ions with the water clusters may arise from two beneficial effects of the water ion beam. First, the water may act as a source of protons aiding in the formation of [M + H]^+^ ions. This theory has been tested by using a (D_2_O)_n_^+^ cluster beam which indicated that proton transfer from the beam to the analyte does occur [35]. Second, the lower E/n of the cluster leads to softer sputtering with reduced fragmentation. This may also be the explanation for the increase in the ion yield of [M + Na/K]^+^ adduct ions. Evidence for the stabilization of these species is provided by the observation in the reduction in the relative intensity of the corresponding trimethylamine, the terminal part of the PC head group, neutral loss (− 59 Da.) fragment ions [M + Na/K-TMA]^+^. In addition, the *m/z* 184 PC head group ion shows no enhancement with increasing cluster size, only a reduction in intensity at lower E/n, also indicating a reduction in fragmentation. The reduced fragmentation is in line with studies of amino acid, arginine, where the ratio of fragments to *pseudo-*molecular ion decreased at lower E/n, and recent studies of benzylpyridinium (BYP) salts that were used to measure a significant reduction in secondary ion internal energy as E/n falls below 0.25 eV [40, 41].

Increasing the cluster size, reducing E/n, may seem an obvious method for softening the ionization in SIMS. However, in practice, the softer sputtering is accompanied by a loss of secondary ion yield. With conventional gas cluster ion beams, even at 20 keV, a reduction in ionization of Irganox 1010 has been shown when switching from Ar_2000_^+^ to Ar_4000_^+^ (E/n = 0.25 and 0.125 respectively) [17]. The water cluster beam is clearly able to mitigate this effect and even enhance the signal beyond that of the higher E/n beams. While it is expected that the water cluster beam may provide a source of protons and lead to increases in [M + H]^+^ ions, mass spectra of compounds such as PC(34:1), cardiolipin salt, and mouse brain tissue show increased Na^+^/K^+^ adduct ion signals and this is evident also on these lipid samples [36, 37]. It has been speculated that the increased cationization comes from the aqueous environment resulting from bombardment of water clusters, but the reduced fragmentation of these species as evidenced by the reduced TMA loss, along with other fragmentation pathways, will also play a role [36].

While the data in Fig. 1 show the optimum conditions for generating intact molecular signals, it is also interesting to consider the relative intensities of these different *pseudo-*molecular ions. As with any MS imaging approach, SIMS signals can be strongly influenced by matrix effects, which in the case of bio-molecules, is often related to the transfer of protons to produce [M ± H]^±^ ions. The relative intensities of the PE lipids detected as [M-H]^−^ ions in the SIMS and the PC lipids detected as [M + H]^+^ ions, in the absence of isobaric interferences, show good agreement with quantitative LC-MS analysis of the same lipid extract sample using ESI ionization (producing [M + H]^+^ ions of both lipid classes) (ESM Fig. S2). This indicates that with lipids, the main problem is comparisons between lipid classes as opposed to within a lipid class.

Supplying a source of protons (and hydroxyl ions) may present an opportunity for reducing the matrix effect and thus improve relative quantitation. Figure 2 shows excerpts of the lipid mass region of mass spectra from analyses of porcine brain lipid extract and bovine heart lipid extract using (H_2_O)_22.5k_^+^ compared to analysis using (CO_2_)_6k_^+^. The base peak in the brain extract spectrum is at *m/z* 888, while the highest peak in the heart spectrum is the *m/z* 861 ion.

Overall enhancements are observed, but not all peaks show the same relative enhancement. Some of the relative enhancement differences may be explained due to the softer ionization. For example, the change in the relative intensity between the prominent *m/z* 701 and *m/z* 788 peaks in the brain lipid spectra arises since the *m/z* 701 peak is assigned as an intense fragment ion of the [PS(36:1) − H]^−^ ion at *m/z* 788.5 [42]. *m/z* 701 is sometimes assigned as a PA(36:1) lipid in SIMS analysis, but here, the strong signal at *m/z* 788 and the relatively small peak at *m/z* 701 in the ESI spectrum (ESM Fig. S3) provide strong evidence for the PS fragment assignment. Sulfatides, such as ST(42:2) at *m/z* 888.6, have been shown to be enhanced in lipid mixtures so an amelioration of any matrix effects may not influence these species as much as others [13].

In the case of the heart lipid extract sample (Fig. 2B), it is the phosphatidylethanolamine (PE) and phosphatidylglycerol (PG) lipids that show the greatest relative enhancement compared to the other lipid classes, most clearly observed for [PE(P-36:2) − H]^−^ at *m/z* 726, [PE(38:4) − H]^−^ at *m/z* 766, and [PG(34:1) − H]^−^ at *m/*z 747 (Fig. 2B and ESM Table S2).

### Secondary ion yield enhancement in lipid extracts when using CO_2_ as backing gas

The data shown in Figs. 1 and 2 was acquired using either pure CO_2_ clusters or pure H_2_O clusters (using a N_2_ backing gas). The effect of the backing gas used when forming the water clusters was also studied. Lipid extracts from porcine brain and bovine heart along with cells from a MCF7 cell line were analyzed using water clusters with two different backing gasses, N_2_, as it ensures pure water clusters [(H_2_O)_n_^+^] and CO_2_ [(H_2_O)_n_^+^ (CO_2_)]. CO_2_ readily forms clusters and is expected to be incorporated into the water cluster, although to what degree is currently unknown. Cluster mass is measured by time-of-flight down the ion beam column to the sample, but there is no means of confirming the precise composition in our laboratory at present. It should be noted that this does not change the E/n in the cluster; however, the incorporation of 1 CO_2_ molecule (44 Da.) would mean that the number of water molecules (18 Da.) in the cluster must have been reduced by a factor of 2.4. However, for simplicity, we state the “pure water” cluster size in all cases and specify the backing gas when CO_2_ was used. For future studies, an approach composition measurement using a residual gas analyzer as demonstrated by Wucher and co-workers may be helpful [43].

Figure 3 shows a spectral overlay comparing the signal between (H_2_O)_n_^+^ clusters and our usual (CO_2_)_n_^+^ cluster beam. The signal from the pure (CO_2_)_n_^+^ cluster beam, pure (H_2_O)_n_^+^ clusters (using N_2_ as a backing gas), and the mixed (H_2_O)_n_^+^ (CO_2_) is shown in orange, light blue, and black, respectively. Figure 3A–D show spectral excerpts of mass spectra acquired from lipid extract analysis. When comparing the signal obtained with (H_2_O)_n_^+^ to (CO_2_)_n_^+^, an increase in secondary ion yield is observed in the data acquired with the water cluster beam. For both samples and ion modes, an enhancement was found when analyzing with the water cluster beam, with the greatest enhancement observed when using pure water clusters. In ESM Table S2, the enhancements when using the (H_2_O)_n_^+^ cluster beams are presented as a ratio compared to the secondary ion yield when analyzing with the (CO_2_)_n_^+^ cluster beam. The ions mentioned in this section are also presented in Table 1. In the analyses of brain lipid extract in negative ion mode, signal enhancement factors of 1.8–2.5 and 1.3–2.4 were found for phospholipids when using (H_2_O)_n_^+^ and (H_2_O)_n_^+^ (CO_2_), respectively. The largest enhancement was found for [PS(36:1) − H]^−^ at *m/z* 788 for both ion beams, likely due to reduced fragmentation as mentioned above. In the analyses of heart lipid extract in negative ion mode, signal enhancement factors of 1.5–4.1 and 0.8–2.1 were found when using (H_2_O)_n_^+^ and (H_2_O)_n_^+^ (CO_2_), respectively. The largest enhancement was found for [PE(P-36:2) − H]^−^ at *m/z* 726, [PE(38:4) − H]^−^ at *m/z* 766, and [PG(34:1) − H]^−^ at *m/z* 747 for both ion beams. In positive ion mode, larger enhancements in the secondary ion yield were found in both samples. In the brain lipid extract analyses, an enhancement factor of 2.1–3.9 for the phospholipids and 15.9–187.3 for the cholesterol species were found when using (H_2_O)_n_^+^. When using the (H_2_O)_n_^+^ (CO_2_) cluster beam, an enhancement factor of 1.9–2.9 for the phospholipids and 11.6–167.8 for the cholesterol species were found. The largest enhancement was found for [Chol. + K]^+^ at *m/z* 425 for both ion beams. For the phospholipids, the largest enhancement was found for [PC(34:1) + Na]^+^ at *m/z* 782. In the heart lipid extract analyses, an enhancement factor of 1.5–2.5 was found for the phospholipids and 11.0 for cholesterol was found when using (H_2_O)_n_^+^. When using the (H_2_O)_n_^+^ (CO_2_) cluster beam, an enhancement factor of 1.0–2.2 was found for the phospholipids and 9.0 for cholesterol. For both ion beams, the largest enhancement was found for [Chol. + H-H_2_O]^+^ at *m/z* 369, while for the phospholipids, the largest enhancement was found for [SM(36:1) + Na]^+^ at *m/z* 753.

**Table 1:** Table of secondary ion yield enhancements when using (H_2_O)_n_^+^ cluster beams over using a (CO_2_)_n_^+^ cluster beam. The table includes information on putative assignments, and ion species, measured *m/z*, and secondary ion yield enhancement with error are presented. Enhancements are presented as a ratio compared to the secondary ion yield when using the (CO_2_)_n_^+^ cluster beam. Error takes into consideration the error of the current measurement for each experiment. This is an excerpt of ESM Table S2

Assignment	Species	Measured *m/z*	(H_2_O)_22.5k_^+^	(H_2_O)_22.5k_^+^ (CO_2_)
			Ion yield enhancement	Ion yield enhancement
Brain lipid extract				
Negative ion mode				
PS(36:1)	[M-H]^−^	788.55	2.48 ± 0.33	2.44 ± 0.61
ST(42:2)	[M-H]^−^	888.62	1.81 ± 0.23	1.27 ± 0.33
Positive ion mode				
Chol.	[M + K]^+^	435.32	187.26 ± 24.56	167.77 ± 42.07
PC(34:1)	[M + Na]^−^	782.57	3.03 ± 0.41	2.59 ± 0.66
Heart lipid extract				
Negative ion mode				
PE(36:2)	[M-H]^−^	726.56	4.10 ± 0.57	1.92 ± 0.51
PG(34:1)	[M-H]^−^	747.53	3.89 ± 0.54	1.95 ± 0.50
PE(38:4)	[M-H]^−^	766.56	3.93 ± 0.53	2.07 ± 0.56
Positive ion mode				
Chol.	[M + H-H_2_O]^+^	369.95	10.98 ± 1.42	9.00 ± 2.28
SM(36:1)	[M + Na]^+^	753.59	2.52 ± 0.32	2.17 ± 0.55

Figure 3A shows how the ratio of *m/z* 701, 788, and 888, mentioned above, changes when using (H_2_O)_n_^+^ (CO_2_) compared to (H_2_O)_n_^+^ and CO_2_^+^. The biggest difference is found with [ST(42:2) − H]^−^ at *m/z* 888 with a secondary ion yield enhancement of 1.8 when using (H_2_O)_n_^+^ and an enhancement of 1.3 when using (H_2_O)_n_^+^ (CO_2_) (Table 1 and ESM Table S2).

To investigate the effect of the backing gas on a more complex sample, cells from a MCF7 cell line were analyzed using the same ion beams. In negative ion mode (Fig. 3E), similar enhancement in secondary ion yield was observed as in the lipid extract analyses, where (H_2_O)_n_^+^ showed higher intensity lipid signals than both (H_2_O)_n_^+^ (CO_2_) and the reference beam of (CO_2_)_n_^+^. In ESM Table S3, the enhancements were calculated as a ratio compared to the secondary ion yield when analyzing with the (CO_2_)_n_^+^ beam. Enhancement factors of 3.7–9.7 and 1.3–3.6 were found when using (H_2_O)_n_^+^ and (H_2_O)_n_^+^ (CO_2_), respectively. For both ion beams, the largest enhancement was found for [PE(36:1) − H]^−^ at *m/z* 742.

Contrary to the other samples analyzed, in positive ion mode (Fig. 3F), a higher secondary ion yield was gained when using the (H_2_O)_n_^+^ (CO_2_) cluster ion beam. Enhancement factors of 1.6–4.3 and 2.7–6.6 were found when using (H_2_O)_n_^+^ and (H_2_O)_n_^+^ (CO_2_), respectively. For both ion beams, the largest enhancement was found for an unidentified ion at *m/z* 703. As mentioned above, it has previously been proposed that the enhancement observed when using water as primary ion source is due to an increased amount of H^+^, which could explain the signal increase found in positive ion mode data.

It can be speculated that using CO_2_ as a backing gas may have a number of different effects. Small clusters of mixed H_2_O/CO_2_ are likely to be formed as has been reported for small clusters (*n* < 20) measured by ToF-MS [44]. It is possible that solvation of the CO_2_ in either the water reservoir or in the water cluster itself may lead to the generation of formic acid. While small formic acid cluster beams have been generated in other studies, including the generation of (HCOOH)_n_(H_2_O)_m_H^+^ [45], proton transfer in H_2_O/CO_2_ beams was not detected when the cluster ionization was performed using single photon ionization at 26.5 eV. If formic acid is produced, it will most likely result in dissociated H^+^, HCO_3_^−^, and CO_3_^2−^ either in the cluster or upon impact with the sample surface at 40 keV.

The results in this section illustrate that the effect of using different ion beams can greatly differ between samples. Subsequently, further investigation is necessary to determine the benefits of using a water cluster and mixed beams on different types of samples.

**Fig. 4:**
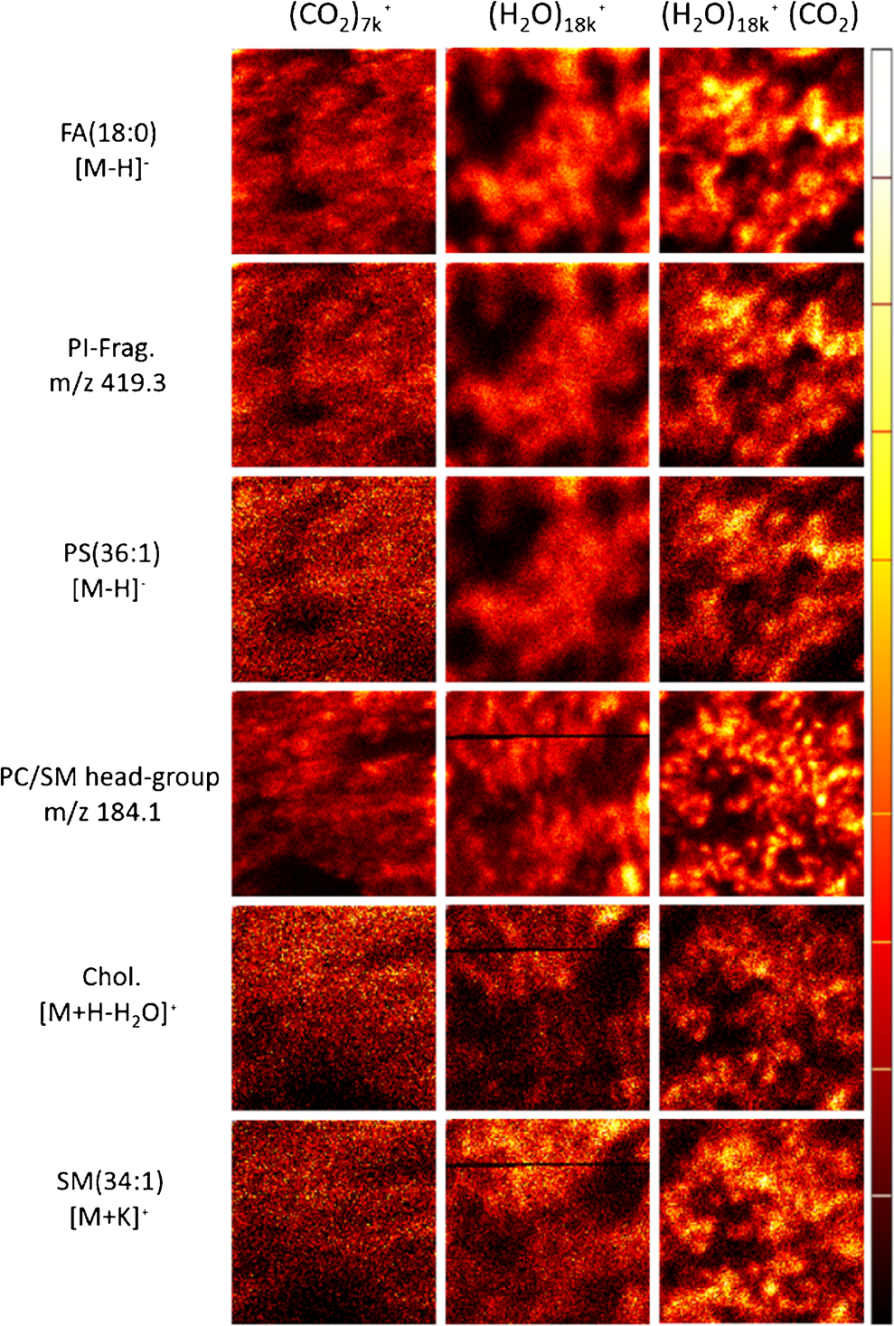
Single ion images of cells analyzed with three different ion beams, (CO_2_)_7k_^+^, (H_2_O)_18_^+^, and (H_2_O)_18_^+^ (CO_2_). Single ion images of [FA:18:0 − H]^−^ (*m/z* 283.3, 0.04 ppm), PI-Frag. (*m/z* 419.3, 0.04 ppm), [PS(36:1) − H]^−^ (*m/z* 788.6, 3.0 ppm), PC/SM head group (*m/z* 184.1, 0.24 ppm), [Chol. + H-H_2_O]^+^ (*m/z* 369.4, 0.09 ppm), and [SM(34:1) + K]^+^ (*m/z* 741.5, 4.7 ppm). Analysis area 600 μm × 600 μm and a fluence of 1 × 10^13^ ions/cm^2^. Images are individually scaled to their maximum intensity to highlight signal distributions

During the cell analyses, it was found that the (H_2_O)_n_^+^ (CO_2_) ion beam appears to produce sharper images compared to both the reference beam of (CO_2_)_n_^+^ and the water beam (H_2_O)_n_^+^. This is shown in Fig. 4. The sharper images were found in both negative and positive ion mode, even though in negative ion mode, the signal intensity was higher in the (H_2_O)_n_^+^ analysis (Fig. 3E). This suggests that depending on the purpose of the experiment, it might be preferential to use (H_2_O)_n_^+^ (CO_2_), even though a higher signal might be obtained by using the (H_2_O)_n_^+^ ion beam, although further measurements on a wider range of samples may be needed to confirm this.

### ToF-SIMS imaging of Alzheimer’s brain tissue

**Fig. 5:**
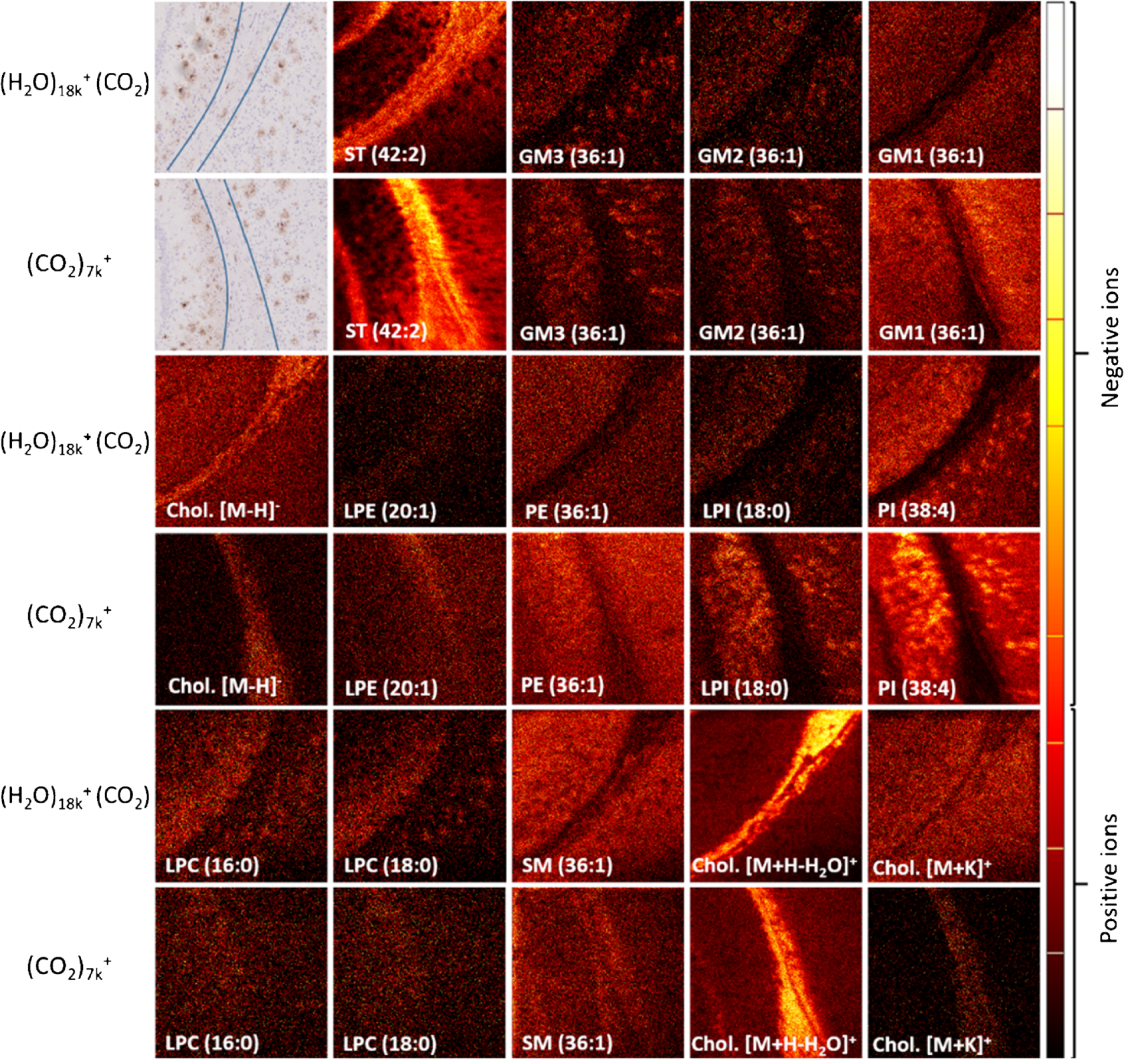
Hematoxylin and β-amyloid-stained images of the areas analyzed with ToF-SIMS and single ion images of various species in the brain tissue. Single ion images of [ST(42:2) − H]^−^ (*m/z* 888.6, 0.04 ppm), [GM3(36:1) − H]^−^ (*m/z* 1179.8, 27.0 ppm), [GM2(36:1) − H]^−^ (*m/z* 1382.9, 37.3 ppm), [GM1(36:1) − H]^−^ (*m/z* 1544.9, 26.9 ppm), [Chol. − H]^−^ (*m/z* 385.4, 8.7 ppm), [LPE(20:1) − H]^−^ (*m/z* 506.3, 7.6 ppm), [PE(36:1) − H]^−^ (*m/z* 744.6, 5.4 ppm), [LPI(18:0) − H]^−^ (599.3, 7.6 ppm), [PI(38:4) − H]^−^ (*m/z* 885.6, 4.3 ppm), [LPC(16:0) + K]^+^ (*m/z* 534.3, 3.2 ppm), [LPC(18:0) + K]^+^ (*m/z* 562.3, 6.6 ppm), [Chol. + H-H_2_O]^+^ (*m/z* 369.4, 0.02 ppm), and [Chol. + K]^+^ (*m/z* 425.3, 3.5 ppm). The three most common isotopes have been added to create the single ion images of GM3, GM2, and GM1. The brightness of the single ion images of GM3, GM2 analyzed with (H_2_O)_18k_^+^ (CO_2_), and LPCs analyzed with (H_2_O)_18k_^+^ (CO_2_) and (CO_2_)_7k_^+^ has been increased by 66%. Analysis area 1250 μm × 1250 μm and a fluence of 2.1 × 10^12^ ions/cm^2^ for (H_2_O)_18k_^+^ (CO_2_) and 7.6 × 10^12^ ions/cm^2^ for (CO_2_)_7k_^+^. Images are individually scaled to their maximum intensity to highlight signal distributions

To confirm the observations obtained from the lipid extracts and cell analyses, as well as to investigate any additional effects when used on an even more complex sample, brain tissue from an Alzheimer’s disease transgenic mouse model was analyzed with (CO_2_)_7k_^+^ and (H_2_O)_18k_^+^ (CO_2_). Figure 5 shows single ion images of a selection of species associated with plaques in Alzheimer’s disease which have previously been imaged with matrix-assisted laser desorption ionization (MALDI) where analysis of consecutive tissue slices from the same animal has been published [46]. Analysis of this well-characterized sample provides a good measure of any shortcomings of SIMS and the potential benefits of water clusters for analysis. The MALDI-MS analysis showed, amongst other things, depletion of sulfatide (ST) species and increase in phosphatidylinositol (PI) species associated with the plaques along with accumulation of specific gangliosides and lysophospholipids. Previous SIMS studies on Alzheimer’s model mouse tissue have also reported sulfatide depletion, but have used pooled signal from multiple peaks to generate the images. Here, both GCIBs show clear ST depletion that can be illustrated using individual [M-H]^−^ species. Similarly, individual PI lipid ions provide sufficient signal to clearly identify the plaques. Imaging intact species over 1000 Da. has been a significant challenge in SIMS for many years; however, as can be seen in Fig. 5, both the (CO_2_)_7k_^+^ and the (H_2_O)_18k_^+^ (CO_2_) cluster beams show localized GM2 and GM3 accumulations on the plaques, while GM1 is more generally associated with the gray matter in this part of the brain. These observations show good correlation with MALDI images from the same animal [46].

In addition, Fig. 5 also shows single ion images of three cholesterol species that are not normally detected in MALDI imaging experiments; [Chol. − H]^−^ at *m/z* 385, [Chol. − H_2_O + H]^+^ at *m/z* 369, and [Chol. + K]^+^ at *m/z* 425. Over the years, there has been a debate over the cholesterol signal in brain tissue in the SIMS community [47–49]. The single ion image of the data acquired using the more conventional (CO_2_)_7k_^+^ ion beam shows how the cholesterol signal is localized to the white matter of corpus callosum (cc). In the single ion image acquired with the (H_2_O)_18k_^+^ (CO_2_) ion beam, while a higher signal is still found in cc, the cholesterol signal is more evenly distributed over the entire analyzed area, i.e., white and gray matter. This is in agreement with a previous study where rodent brain tissue was analyzed with a water cluster ion beam [36]. In the case of the cationic cholesterol species at *m/z* 369, a 20-fold increase of the signal was observed in white matter and a 100–200-fold increase in gray matter. It was hypothesized that the addition of water to the tissue reduces potential matrix effects due to competition for protons [36]. Our data shows a 10-fold and an 8-fold increase for the cholesterol species at *m/z* 369 in the white and gray matter, respectively, when switching from the (CO_2_)_7k_^+^ cluster beam to (H_2_O)_18k_^+^ (CO_2_). The largest signal increase was found in the [M + K]^+^ species at *m/z* 425 with a 15-fold and a 164-fold increase in white and gray matter, respectively. This again shows how the use of a water cluster ion beam does not only benefit the production of [M + H]^+^ species, but also other ion adducts.

Cholesterol has previously been associated with amyloid-beta deposits in Alzheimer’s disease transgenic mouse brains [50]. In the four cholesterol species shown in Fig. 5, it can be seen how, even though other species are detected in the plaques (i.e., GM3, GM2, PI(38:4), LPI(18:0), LPC(16:0), and LPC(18:0)), none of the cholesterol species is localized to the amyloid-beta plaques, clearly depicted in the hematoxylin and β-amyloid-stained images as brown dots and also showing high signal intensity of GM3.

Although previously SIMS has struggled to image LPCs, it is now possible to detect localized LPCs in mouse brain tissue by using the water beam, as is shown in Fig. 5. Excerpts from the total mass spectra from analyses using (CO_2_)_7k_^+^ and (H_2_O)_18k_^+^ (CO_2_) are shown in ESM Fig. S4. LPCs are often detected with MALDI and nano-DESI in diseased or damaged tissue [38, 51, 52]. Now ToF-SIMS is able to do the same, illustrating how the ToF-SIMS results are now more similar to MALDI, with regard to the species that are detected, while maintaining a high lateral resolution and native state of the sample (i.e., no need for sample treatment such as adding a matrix). It is noteworthy that the LPC signals that localize to the plaques are attributed to [M + K]^+^ ions and not to [M + H]^+^ ions. [LPC(16:0) + H]^+^ is observed in the ToF-SIMS spectra and is more prominent when using the (H_2_O)_18k_^+^ cluster beam compared to (CO_2_)_7k_^+^. However, no localization of this ion signal to the plaques was observed. We hypothesize that the [M + H]^+^ ion signal is diluted by substantial contribution from fragmentation of [M + H]^+^ ions of PC lipids. [M + K]^+^ ions of PC lipids preferentially fragment via routes that do not produce isobaric interferences with LPCs (loss of TMA for example) and so have a lower chemical background signal [53, 54]. As with the cell imaging example, the images appear slightly sharper when using the water beam, possibly due to a more stable cluster being produced that is less likely to fragment as it travels down the primary ion gun. Previous improvements in achievable resolution for cluster imaging have also been observed when switching from pure Ar_n_ to CO_2_ containing gas cluster ion beams [55].

## Conclusions

The data provides further evidence of the potential benefits for water cluster ion beams for SIMS analysis, with potential for additional benefits observed when doping the water clusters with carbon dioxide, possibly introducing additional acid-base reactions at the impact site. The relative signal levels of different lipids within the same class were unchanged and, in the case of PCs and PEs, were similar to the ratios in the ESI data. However, the lipid ratios between lipid classes were altered when using the (H_2_O)_n_^+^ ion beam compared to the more conventional (CO_2_)_n_^+^ ion beam, opening up for the question of water’s ability to circumvent matrix effects. In addition, using a GCIB, the localization of phosphatidylinositols and gangliosides, along with the depletion of sulfatide ST(42:2), was successfully imaged at amyloid-beta plaque sites. Despite the considerable increase in several different cholesterol-specific ions, no accumulation on the plaques was observed in this case. While the use of the water clusters results in softer ionization, fragmentation is still a feature of the SIMS analysis. Despite this, LPC accumulation at plaque sites could be detected using [M + K]^+^ ions.

## Supplementary information

ESM 1 (PDF 994 kb)
