# Supplementary material for: (CO2)n+, (H2O)n+, and (H2O)n+ (CO2) gas cluster ion beam secondary ion mass spectrometry: analysis of lipid extracts, cells, and Alzheimer’s model mouse brain tissue
Source: Anal Bioanal Chem. 2021 May 11;413(16):4181–94. doi: 10.1007/s00216-021-03372-x (PMC8222020; doi:10.1007/s00216-021-03372-x)
Supplement: Supplementary file 1 — (PDF 994 kb) [file 216_2021_3372_MOESM1_ESM.pdf]

$(CO_2)_n^+$, $(H_2O)_n^+$, and $(H_2O)_n^+(CO_2)$ gas cluster ion beam secondary ion mass spectrometry: analysis of lipid extracts, cells, and Alzheimer's model mouse brain tissue

Kelly Dimovska Nilsson[1], Anthi Karagianni[1], Ibrahim Kaya[1,2], Marcus Henricsson[3], John S. Fletcher[1*]

1. Department of Chemistry and Molecular Biology, University of Gothenburg, Gothenburg, Sweden
2. Department of Psychiatry and Neurochemistry, Sahlgrenska Academy at the University of Gothenburg, Mölndal, Sweden
3. Department of Molecular and Clinical Medicine/Wallenberg Laboratory, Institute of Medicine, University of Gothenburg, Gothenburg, Sweden

*Corresponding author e-mail: john.fletcher@chem.gu.se

# current addresses (Ibrahim Kaya): Medical Mass Spectrometry Imaging, Department of Pharmaceutical Biosciences, Uppsala University, Uppsala, Sweden

Abstract

This work assesses the potential for new water cluster-based ion beams for improving the capabilities of secondary ion mass spectrometry (SIMS) for in situ lipidomics. The effect of water clusters was compared to carbon dioxide clusters, along with the effect of using pure water clusters compared to mixed water and carbon dioxide clusters. An increased signal was found when using pure water clusters. However, when analyzing cells, a more substantial signal increase was found in positive ion mode when the water clusters also contained carbon dioxide, suggesting that additional reactions are in play. The effects of using a water primary ion beam on a more complex sample were investigated by analyzing brain tissue from an Alzheimer´s disease transgenic mouse model. The results indicate that ToF-SIMS results are approaching those from MALDI as ToF-SIMS was able to image lyso-phosphocholine (LPC) lipids, a lipid class that for a long time has eluded detection during SIMS analyses. Gangliosides, sulfatides and cholesterol were also imaged.

Alzheimer’s mouse brain tissue

Negative ion mode

Measured m/z	Exact m/z	Mass accuracy (ppm)	Label	Species	Formula
385.35	385.35	8.69	Chol.	$[M-H]^-$	$C_{27}H_{45}O$
506.29	506.32	-67.21	LPE(20:1)	$[M-H]^-$	$C_{25}H_{49}NO_7P$
599.32	599.32	-7.58	LPI(18:0)	$[M-H]^-$	$C_{27}H_{52}O_{12}P$
744.56	744.55	5.43	PE(36:1)	$[M-H]^-$	$C_{41}H_{79}NO_8P$
885.55	885.55	4.34	PI(38:4)	$[M-H]^-$	$C_{47}H_{82}O_{13}P$
888.62	888.62	-0.04	ST(42:2)	$[M-H]^-$	$C_{48}H_{90}SNO_{11}$
1179.77	1179.74	27.01	GM3(36:1)	$[M-H]^-$	$C_{59}H_{107}N_2O_{21}$
1382.87	1382.82	37.28	GM2(36:1)	$[M-H]^-$	$C_{67}H_{120}N_3O_{26}$
1544.91	1544.87	26.87	GM1(36:1)	$[M-H]^-$	$C_{73}H_{130}N_3O_{31}$

Positive ion mode

Measured m/z	Exact m/z	Mass accuracy (ppm)	Label	Species	Formula
369.35	369.35	-0.02	Chol.	$[M+H-H2O]^+$	$C_{27}H_{45}$
425.32	425.32	3.52	Chol.	$[M+K]^+$	$C_{27}H_{46}OK$
534.29	534.30	-3.24	LPC(16:0)	$[M+K]^+$	$C_{24}H_{50}NO_7PK$
562.32	562.33	-6.58	LPC(18:0)	$[M+K]^+$	$C_{26}H_{54}NO_7PK$
769.56	769.56	-2.85	SM(36:1)	$[M+K]^+$	$C_{41}H_{83}N2O_6PK$

Cells

Negative ion mode

Measured m/z	Exact m/z	Mass accuracy (ppm)	Label	Species	Formula
283.26	283.26	-0.04	FA(18:0)	$[M-H]^-$	$C_{18}H_{35}O_2$
419.26	419.26	-0.04	PI-Frag.		$C_{21}H_{40}O_6P$
673.49	673.48	6.42	PA(34:1)	$[M-H-74]^-$	$C_{37}H_{70}O_8P$
673.49	673.48	6.42	PG(34:1)	$[M-H]^-$	$C_{37}H_{70}O_8P$
701.51	701.51	1.72	PS-Frag.	$[M-H-87]^-$	$C_{39}H_{74}O_8P$
716.53	716.52	2.94	PC(32:1)	$[M-CH3]^-$	$C_{39}H_{75}NO_8P$
742.54	742.54	2.53	PE(36:1)	$[M-H]^-$	$C_{41}H_{77}NO_8P$
760.51	760.51	2.76	PS(34:1)	$[M-H]^-$	$C_{40}H_{75}NO_{10}P$
788.55	788.54	2.99	PS(36:1)	$[M-H]^-$	$C_{42}H_{79}NO_{10}P$
835.54	835.53	3.79	PI(34:1)	$[M-H]^-$	$C_{43}H_{80}O_{13}P$
863.57	863.56	1.96	PI(36:1)	$[M-H]^-$	$C_{45}H_{84}O_{13}P$
885.55	885.55	-0.04	PI(38:4)	$[M-H]^-$	$C_{47}H_{82}O_{13}P$

Positive ion mode

Measured m/z	Exact m/z	Mass accuracy (ppm)	Label	Species	Formula
184.07	184.07	0.24	PC/SM Head-group frag.		$C_5H_{15}NO_4P$
369.35	369.35	0.09	Chol.	$[M+H-H_2O]^+$	$C_{27}H_{45}$
682.45					
703.58					
732.55	732.55	-0.61	PC(32:1)	$[M+H]^+$	$C_{40}H_{78}NO_8PH$
741.53	741.53	-4.70	SM(34:1)	$[M+K]^+$	$C_{39}H_{79}N_2O_6PK$
760.58	760.59	-12.36	PC(34:1)	$[M+H]^+$	$C_{42}H_{82}NO_8PH$
770.51	770.51	0.89	PC(32:1)	$[M+K]^+$	$C_{40}H_{78}NO_8PK$
798.54	798.54	0.05	PC(34:1)	$[M+K]^+$	$C_{42}H_{82}NO_8PK$
824.55	824.56	-3.93	PC(36:2)	$[M+K]^+$	$C_{44}H_{84}NO_8PK$

851.64	851.64	-2.52	SM(42:2)	$[M+K]^+$	$C_{47}H_{93}N_2O_6PK$
Porcine brain lipid extract					
Negative ion mode					
Measured m/z	Exact m/z	Mass accuracy (ppm)	Label	Species	Formula
385.35	385.35	-0.50	Chol.	$[M-H]^-$	$C_{27}H_{45}O$
701.52	701.51	16.02	PS-Frag.	$[M-H-87]^-$	$C_{39}H_{74}O_8P$
726.56	726.54	21.15	PE(P-36:2)	$[M-H]^-$	$C_{41}H_{77}NO_7P$
766.54	766.54	4.30	PE(38:4)	$[M-H]^-$	$C_{43}H_{77}NO_8P$
788.55	788.54	7.90	PS(36:1)	$[M-H]^-$	$C_{42}H_{79}NO_{10}P$
885.55	885.55	-2.09	PI(38:4)	$[M-H]^-$	$C_{47}H_{82}O_{13}P$
888.62	888.62	-0.02	ST(42:2)	$[M-H]^-$	$C_{48}H_{90}SNO_{11}$
906.63	906.63	-7.32	ST(42:2) (OH)	$[M-H]^-$	$C_{48}H_{92}NO_{12}S$
1544.86	1544.87	-8.34	GM1(36:1)	$[M-H]^-$	$C_{73}H_{130}N_3O_{31}$
1572.88	1572.90	-11.54	GM1(38:1)	$[M-H]^-$	$C_{75}H_{134}N_3O_{31}$
Positive ion mode					
Measured m/z	Exact m/z	Mass accuracy (ppm)	Label	Species	Formula
184.07	184.07	-0.14	PC/SM Head-group frag.		$C_5H_{15}NO_4P$
224.10	224.11	-9.52	PC-Frag.		$C_8H_{18}NO_4PH$
369.35	369.35	-0.05	Chol.	$[M+H-H_2O]^+$	$C_{27}H_{45}$
409.34	409.34	0.69	Chol.	$[M+Na]^+$	$C_{27}H_{46}ONa$
425.32	425.32	2.11	Chol.	$[M+K]^+$	$C_{27}H_{46}OK$
577.52	577.52	4.30	DAG(34:1)	$[M-OH]^+$	$C_{37}H_{69}O_4$
599.50	599.50	3.08	PC(34:1)	$[M+Na-PC]^+$	$C_{37}H_{68}O_4Na$
627.53	627.54	-1.56	DAG(38:4)	$[M-OH]^+$	$C_{41}H_{71}O_4$
697.47	697.48	-9.56	PC(32:0)	$[M+Na-TMA]^+$	$C_{37}H_{71}O_8PNa$
713.46	713.45	4.00	PC(32:0)	$[M+K-TMA]^+$	$C_{37}H_{71}O_8PK$
723.49	723.49	-3.79	PC(34:1)	$[M+Na-TMA]^+$	$C_{39}H_{73}O_8PNa$
734.58	734.57	7.39	PC(32:0)	$[M+H]^+$	$C_{40}H_{80}NO_8PH$
739.47	739.47	0.91	PC(34:1)	$[M+K-TMA]^+$	$C_{39}H_{73}O_8PK$
756.55	756.55	0.36	PC(32:0)	$[M+Na]^+$	$C_{40}H_{80}NO_8PNa$
760.59	760.59	5.67	PC(34:1)	$[M+H]^+$	$C_{42}H_{82}NO_8PH$
772.53	772.53	4.03	PC(32:0)	$[M+K]^+$	$C_{40}H_{80}NO_8PK$
782.57	782.57	-0.09	PC(34:1)	$[M+Na]^+$	$C_{42}H_{82}NO_8PNa$
798.54	798.54	1.75	PC(34:1)	$[M+K]^+$	$C_{42}H_{82}NO_8PK$
810.60	810.60	2.00	PC(36:1)	$[M+Na]^+$	$C_{44}H_{86}NO_8PNa$
826.58	826.57	6.33	PC(36:1)	$[M+K]^+$	$C_{44}H_{86}NO_8PK$
Bovine heart lipid extract					
Negative ion mode					
Measured m/z	Exact m/z	Mass accuracy (ppm)	Label	Species	Formula
385.35	385.35	10.06	Chol.	$[M-H]^-$	$C_{27}H_{45}O$
715.59	715.58	17.11	SM(D36:1)	$[M-CH3]^-$	$C_{40}H_{80}N_2O_6P$
726.56	726.54	20.22	PE(P-36:2)	$[M-H]^-$	$C_{41}H_{77}NO_7P$
747.53	747.52	18.15	PG(34:1)	$[M-H]^-$	$C_{40}H_{76}O_{10}P$
766.53	766.54	-4.79	PE(38:4)	$[M-H]^-$	$C_{43}H_{77}NO_8P$
788.55	788.54	6.35	PS(36:1)	$[M-H]^-$	$C_{42}H_{79}NO_{10}P$
861.55	861.55	5.75	PI(36:2)	$[M-H]^-$	$C_{45}H_{82}O_{13}P$
885.55	885.55	0.02	PI(38:4)	$[M-H]^-$	$C_{47}H_{82}O_{13}P$
1447.98	1447.96	13.23	CL(72:8)	$[M-H]^-$	$C_{81}H_{141}O_{17}P_2$

1469.97	1469.95	14.06	CL(72:8)	$[M-2H+Na]^-$	$C_{81}H_{140}O_{17}P_2Na$

Positive ion mode					
Measured m/z	Exact m/z	Mass accuracy (ppm)	Label	Species	Formula
184.07	184.07	-0.14	PC/SM Head-group frag.		$C_5H_{15}NO_4P$
224.10	224.11	-10.91	PC-Frag.		$C_8H_{18}NO_4PH$
369.35	369.35	-0.07	Chol.	$[M+H-H_2O]^+$	$C_{27}H_{45}$
542.49	542.49	-2.31	SM-Frag		$C_{34}H_{65}NO_2Na$
570.52					
597.49	597.49	6.49	PC(34:2)	$[M+Na-PC]^+$	$C_{37}H_{66}O_4Na$
626.58					
640.60					
694.52	694.52	4.57	SM(36:1)	$[M+Na-TMA]^+$	$C_{38}H_{74}NO_6PNa$
705.48	705.48	-6.91	PC(O-34:3)	$[M+Na-TMA]^+$	$C_{39}H_{71}O_7PNa$
721.47	721.46	22.03	PC(O-34:3)	$[M+K-TMA]^+$	$C_{39}H_{71}O_7PK$
721.47	721.48	-7.04	PC(34:2)	$[M+Na-TMA]^+$	$C_{39}H_{71}O_8PNa$
737.48	737.45	33.62	PC(34:2)	$[M+K-TMA]^+$	$C_{39}H_{71}O_8PK$
742.57	742.58	-8.09	PC(O-34:3)	$[M+H]^+$	$C_{42}H_{81}NO_7P$
753.59	753.59	2.32	SM(36:1)	$[M+Na]^+$	$C_{41}H_{83}N_2O_6PNa$
758.57	758.57	-2.00	PC(34:2)	$[M+H]^+$	$C_{42}H_{81}NO_8P$
764.55	764.56	-3.18	PC(O-34:3)	$[M+Na]^+$	$C_{42}H_{80}NO_7PNa$
780.55	780.53	26.83	PC(O-34:3)	$[M+K]^+$	$C_{42}H_{80}NO_7PK$
780.55	780.55	-0.04	PC(34:2)	$[M+Na]^+$	$C_{42}H_{80}NO_8PNa$
796.55	796.53	32.75	PC(34:2)	$[M+K]^-$	$C_{42}H_{80}NO_8PK$
809.64	809.65	-17.92	SM(40:1)	$[M+Na]^+$	$C_{45}H_{91}N_2O_6PNa$
823.67	823.67	3.57	SM(41:1)	$[M+Na]^+$	$C_{46}H_{93}N_2O_6PNa$
837.69	837.68	3.76	SM(42:1)	$[M+Na]^+$	$C_{47}H_{95}N_2O_6PNa$

Table S1. Table of species mentioned in this work. Measured mass, calculated exact mass, mass accuracy, label, and formula of each species are presented. Green: species used as calibration points.

Porcine brain lipid extract

Negative ion mode

Assignment	Species	Measured m/z	$(H2O)_{22.5k}^{+}$ Ion yield enhancement	$(H2O)_{22.5k}^{+}(CO_2)$ Ion yield enhancement
PS-Frag.	[M-H-87]-	701.52	1.82 ± 0.24	1.68 ± 0.42
PE(P-36:2)	[M-H]-	726.56	2.10 ± 0.30	1.38 ± 0.35
PS(36:1)	[M-H]-	788.55	2.48 ± 0.33	2.44 ± 0.61
ST(42:2)	[M-H]-	888.62	1.81 ± 0.23	1.27 ± 0.33
ST(42:2) (OH)	[M-H]-	906.63	2.20 ± 0.28	1.68 ± 0.45
GM1(36:1)	[M-H]-	1544.64	2.27 ± 0.29	1.78 ± 0.45
GM1(38:1)	[M-H]-	1572.66	2.26 ± 0.28	1.80 ± 0.45

Positive ion mode

Assignment	Species	Measured m/z	$(H2O)_{22.5k}^{+}$ Ion yield enhancement	$(H2O)_{22.5k}^{+}(CO_2)$ Ion yield enhancement
Chol.	[M+H-H2O]$^+$	369.35	15.89 ± 2.12	11.63 ± 2.91
Chol.	[M+Na]$^+$	409.34	60.43 ± 7.90	51.29 ± 12.91
Chol.	[M+K]$^+$	425.32	187.26 ± 24.56	167.77 ± 42.07
DAG(34:1)	[M-OH]$^+$	577.52	2.42 ± 0.32	2.14 ± 0.55
PC(34:1)	[M+Na-PC]$^+$	599.5	2.15 ± 0.28	1.93 ± 0.49
DAG(38:4)	[M-OH]$^+$	627.53	2.11 ± 0.30	1.85 ± 0.48
PC(34:1)	[M+K-TMA]$^+$	739.47	2.30 ± 0.32	1.89 ± 0.48
PC(34:1)	[M+H]$^+$	760.59	3.92 ± 0.53	2.91 ± 0.74
PC(34:1)	[M+Na]$^+$	782.57	3.03 ± 0.41	2.59 ± 0.66
PC(34:1)	[M+K]$^+$	798.55	2.91 ± 0.40	2.41 ± 0.61
PC(36:1)	[M+Na]$^+$	810.6	2.96 ± 0.41	2.46 ± 0.63
PC(36:7)	[M+Na]$^+$	826.58	2.93 ± 0.41	2.39 ± 0.61

Bovine heart lipid extract

Negative ion mode

Assignment	Species	Measured m/z	$(H2O)_{22.5k}^{+}$ Ion yield enhancement	$(H2O)_{22.5k}^{+}(CO_2)$ Ion yield enhancement
SM(36:1)	[M-CH3]$^-$	715.59	2.63 ± 0.41	1.38 ± 0.35
PE(P-36:2)	[M-H]$^-$	726.56	4.10 ± 0.57	1.92 ± 0.51
PG(34:1)	[M-H]$^-$	747.53	3.89 ± 0.54	1.95 ± 0.50
PE(38:4)	[M-H]$^-$	766.56	3.93 ± 0.53	2.07 ± 0.56
PI(36:2)	[M-H]$^-$	861.55	2.19 ± 0.32	1.09 ± 0.28
PI(38:4)	[M-H]$^-$	885.55	1.88 ± 0.27	0.94 ± 0.24
CL(72:8)	[M-H]$^-$	1447.68	1.50 ± 0.24	0.84 ± 0.23
CL(72:8)	[M-2H+Na]$^-$	1469.64	1.45 ± 0.20	0.76 ± 0.21

Positive ion mode

Assignment	Species	Measured m/z	$(H2O)_{22.5k}^{+}$ Ion yield enhancement	$(H2O)_{22.5k}^{+}(CO_2)$ Ion yield enhancement
Chol.	[M+H-H2O]$^+$	369.35	10.98 ± 1.42	9.00 ± 2.28
SM-Frag		542.49	2.00 ± 0.25	1.53 ± 0.38
		570.52	2.02 ± 0.26	1.59 ± 0.40

PC(34:2)	$[M+Na-PC]^+$	597.49	1.51 ± 0.20	1.00 ± 0.26
		626.58	2.00 ± 0.25	1.57 ± 0.40
		640.6	2.06 ± 0.26	1.60 ± 0.40
SM(36:1)	$[M+Na-TMA]^+$	694.52	2.02 ± 0.26	1.51 ± 0.38
SM(36:1)	$[M+Na]^+$	753.59	2.52 ± 0.32	2.17 ± 0.55
SM(41:1)	$[M+Na]^+$	764.57	1.82 ± 0.23	1.35 ± 0.34
PC(34:2)	$[M+K]^+$	780.55	1.92 ± 0.25	1.32 ± 0.34
SM(40:1)	$[M+Na]^+$	809.64	2.36 ± 0.30	2.02 ± 0.51
SM(41:1)	$[M+Na]^+$	823.67	2.37 ± 0.31	2.02 ± 0.51
SM(42:1)	$[M+Na]^+$	837.69	2.49 ± 0.31	2.06 ± 0.52

Table S2. Table of secondary ion yield enhancements when using $(H2O)_n^+$ cluster beams over using a $(CO_2)^+$ cluster beam. Assignment, measured m/z, secondary ion yield enhancement and error are presented. Enhancements are presented as a ratio compared to the secondary ion yield when using CO_2 ion beam. Error takes into consideration the error of the current measurement for each experiment.

Cells				
Negative ion mode				
Assignment	Species	Measured m/z	$(H2O)_{18k}^{+}$	$(H2O)_{18k}^{+}$ (CO_2)
			Ion yield	Ion yield
PA(34:1)	[M-H-74]-	673.49	4.61	1.73
PG(34:1)	[M-H]-	673.49	4.61	1.73
PS-Frag.	[M-H-87]-	701.51	4.7	1.79
PC(32:1)	[M-CH3]-	716.53	9.29	3.33
PE(36:1)	[M-H]-	742.54	9.67	3.6
PE(38:7)	[M-H]-	760.51	8.75	2.58
PS(36:1)	[M-H]-	788.55	8.48	2.58
PI(34:1)	[M-H]-	835.54	4.1	1.51
PI(36:1)	[M-H]-	863.57	3.67	1.36
PI(38:4)	[M-H]-	885.55	4.14	1.33
Positive ion mode				
Assignment	Species	Measured m/z	$(H2O)_{18k}^{+}$	$(H2O)_{18k}^{+}$ (CO_2)
			Ion yield	Ion yield
		682.45	1.59	2.68
		703.58	4.27	6.57
PC(32:1)	[M+H]+	732.55	4.23	5.86
SM(34:1)	[M+K]+	741.53	2.74	4.44
PC(34:1)	[M+H]+	760.58	3.9	5.57
PC(32:1)	[M+K]+	770.51	3.17	5.04
PC(34:1)	[M+K]+	798.54	2.71	4.26
PC(36:2)	[M+K]+	824.55	3.14	4.5
SM(42:2)	[M+K]+	851.64	2.5	4.37

Table S3. Table of secondary ion yield enhancements when using $(H2O)_n^+$ beams over using a $(CO_2)_n^+$ cluster ion beam. Assignment, measured m/z and secondary ion yield enhancement are presented. Enhancements are presented as a ratio compared to the secondary ion yield when using $(CO_2)_n^+$ cluster ion beam.

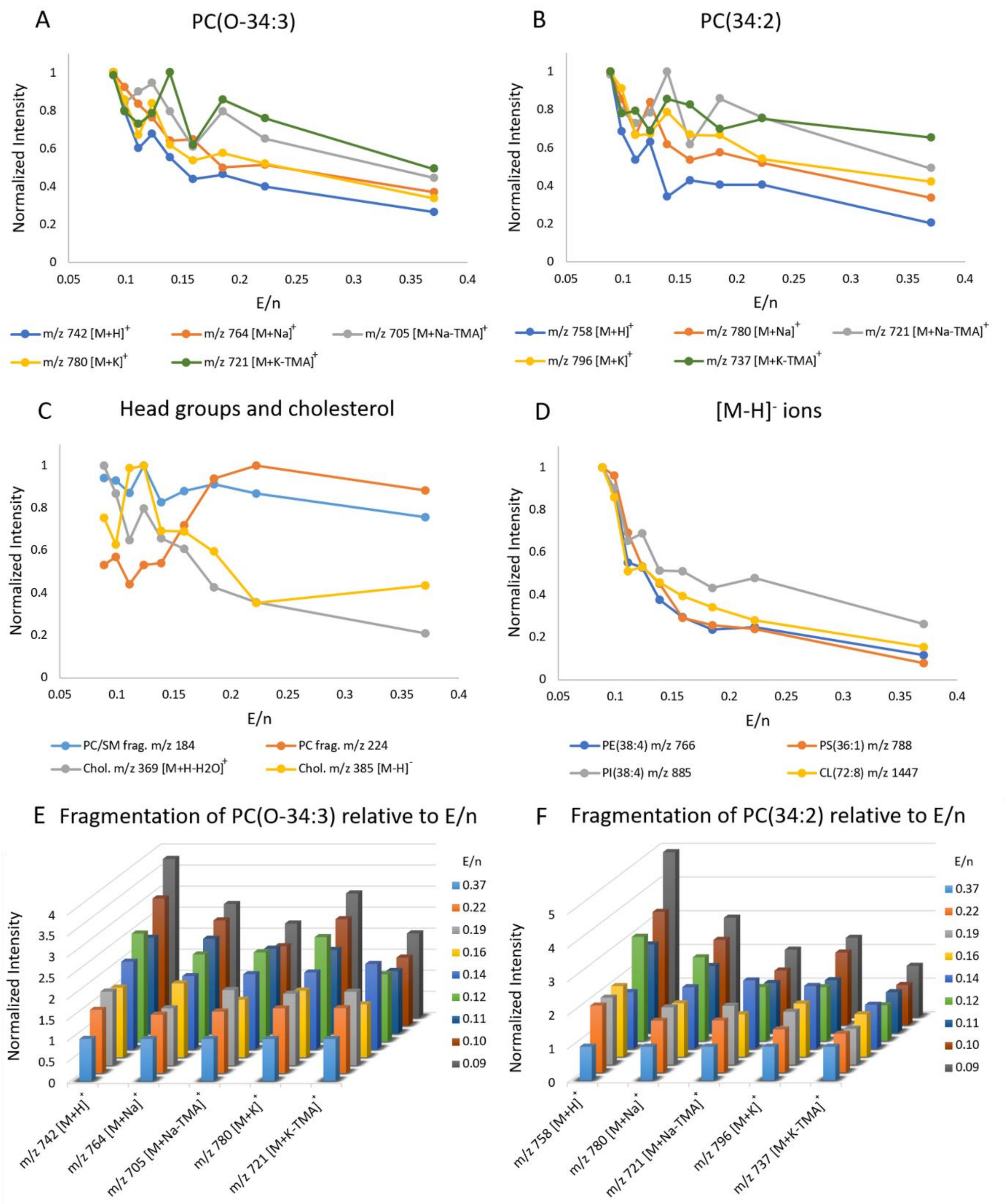

Fig. S1. A-D: Plots of various species from analysis of bovine heart lipid extract. Shown is signal intensity relative to cluster size (E/n) with cluster sizes varying from 6000 to 22500. Signal is normalized to maximal signal. E&F: Bar charts showing reduced fragmentation of various species with lower E/n. Signal is normalized to the signal using cluster size 6000. Analysis area 200 µm × 200 µm with a primary ion dose density of 2.56×10^{12} ions/cm^2.

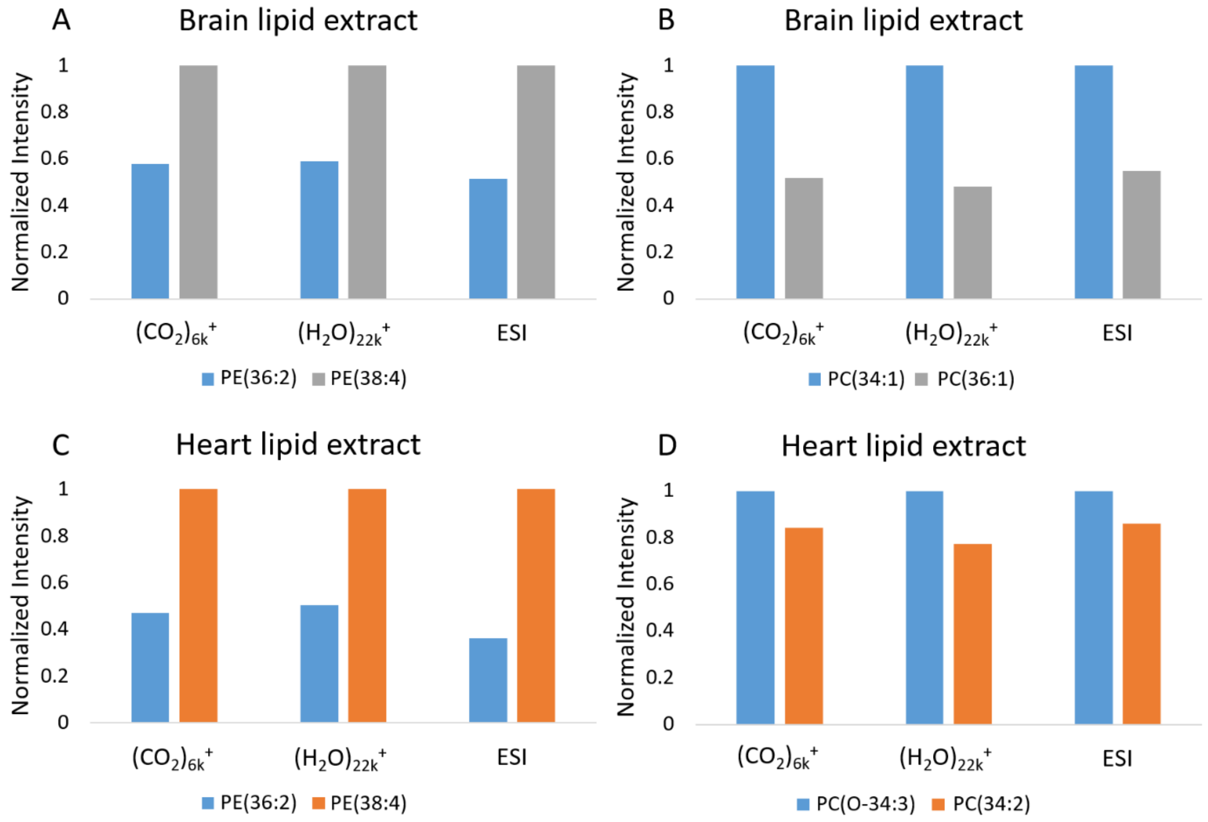

Fig. S2. Bar charts comparing the intensities between SIMS analysis and ESI analysis. PE(36:2) at m/z 742.6, PE(38:4) at m/z 766.5, PC(34:1) at m/z 760.6 and PC(36:1) at m/z 788.6 from porcine brain lipid extract are plotted. PE(36:2) at m/z 742.5, PE(38:4) at m/z 766.5, PC(O-34:3) at m/z 742.6 and PC(34:2) at m/z 758.6 from bovine heart lipid extract are plotted. A & C acquired in negative ion more, B & D acquired in positive ion mode. Intensities normalized to highest intensity.

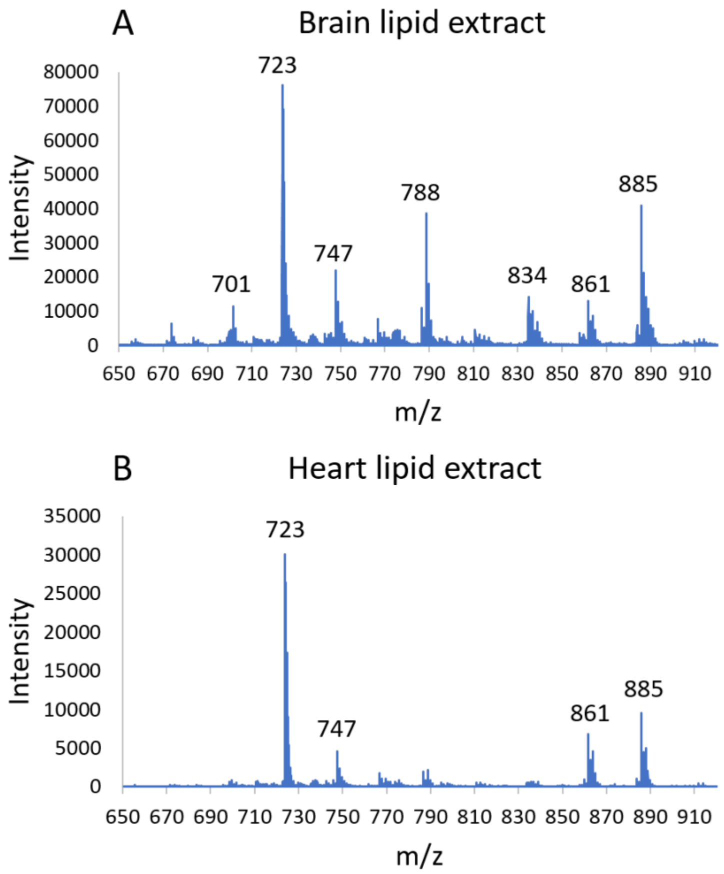

Fig. S3. A: Excerpts of Synapt G2 mass spectra from ESI analysis of porcine brain lipid extract, B: Excerpts of mass spectra from ESI analysis of bovine heart lipid extract.

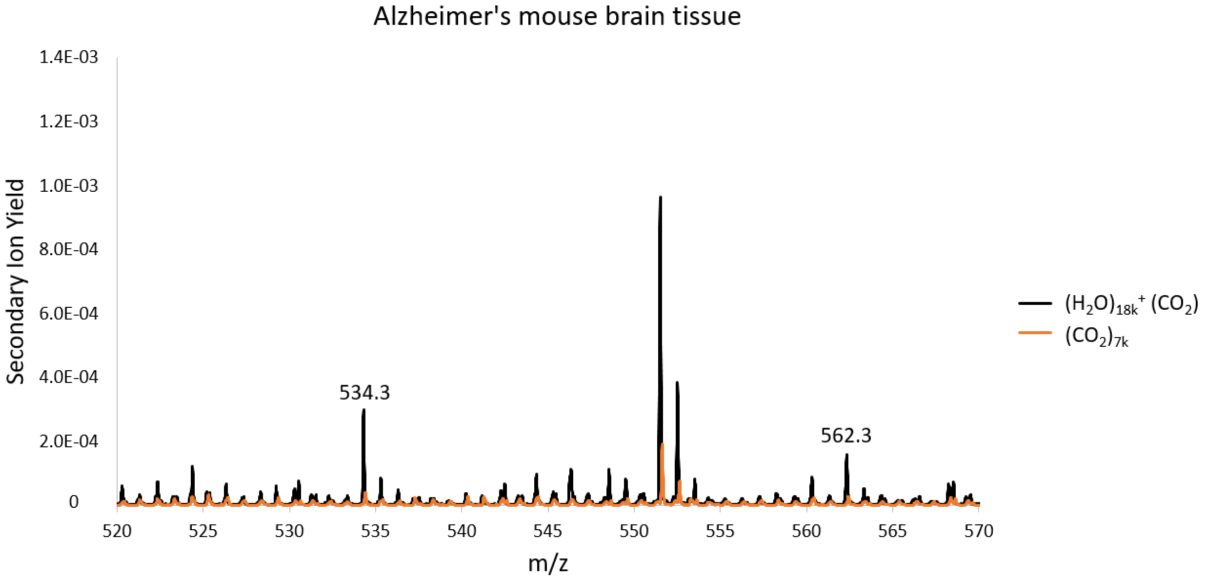

Fig. S4. Excerpts from the total mass spectra from analysis of Alzheimer's mouse brain tissue analyzed using $(CO_2)_{7k}^+$ and $(H_2O)_{18k}^+$ (CO_2). Shown is the secondary ion yield of $[LPC(16:0) + K]^+$ (m/z 534.3, 3.2 ppm) and $[LPC(18:0) + K]^+$ (m/z 562.3, 6.6 ppm). Analysis area 1250 µm × 1250 µm and a fluence of 2.1×10^{12} ions/cm^2 for $(H_2O)_{18k}^+$ (CO_2) and 7.6×10^{12} ions/cm^2 for $(CO_2)_{7k}^+$.
